# Protecting the non-operative lobe/s of the operative lung can reduce the pneumonia incidence after thoracoscopic lobectomy: a randomised controlled trial

**DOI:** 10.1038/s41598-024-60114-6

**Published:** 2024-04-24

**Authors:** Chao Zhou, Shan Song, Jianfeng Fu, Xuelian Zhao, Huaqin Liu, Huanshuang Pei, Shasha Zhang, Hongbo Guo, Xinxin Cui

**Affiliations:** 1 Department of Anesthesiology, The Fourth Hospital of Hebei Medical University, Shijiazhuang, Hebei China; 2 Department of Respiratory, The Fourth Hospital of Hebei Medical University, Shijiazhuang, Hebei China; 3 Department of Anesthesiology, The Fourth Hospital of Shijiazhuang, Shijiazhuang, Hebei China

**Keywords:** Bronchial blocker, Continuous positive airway pressure, Lung isolation, Pneumonia, Thoracoscopic lobectomy, Randomized controlled trials, Risk factors

## Abstract

Lung isolation usually refers to the isolation of the operative from the non-operative lung without isolating the non-operative lobe(s) of the operative lung. We aimed to evaluate whether protecting the non-operative lobe of the operative lung using a double-bronchial blocker (DBB) with continuous positive airway pressure (CPAP) could reduce the incidence of postoperative pneumonia. Eighty patients were randomly divided into two groups (n = 40 each): the DBB with CPAP (Group DBB) and routine bronchial blocker (Group BB) groups. In Group DBB, a 7-Fr BB was placed in the middle bronchus of the right lung for right lung surgery and in the inferior lobar bronchus of the left lung for left lung surgery. Further, a 9-Fr BB was placed in the main bronchus of the operative lung. In Group BB, routine BB placement was performed on the main bronchus on the surgical side. The primary endpoint was the postoperative pneumonia incidence. Compared with Group BB, Group DBB had a significantly lower postoperative pneumonia incidence in the operative (27.5% vs 5%, *P* = 0.013) and non-operative lung (40% vs 15%) on postoperative day 1. Compared with routine BB use for thoracoscopic lobectomy, using the DBB technique to isolate the operative lobe from the non-operative lobe(s) of the operative lung and providing CPAP to the non-operative lobe(s) through a BB can reduce the incidence of postoperative pneumonia in the operative and non-operative lungs.

## Background

Double-lumen tubes (DLT) and bronchial blockers (BB) are the most commonly used airway tools in thoracic surgery^1^. They allow isolation of the operative lung from the non-operative lung, which prevents contamination of the non-operative lung by pollutants, such as blood and sputum^2^. However, in thoracoscopic lobectomy, these two options only protect the non-operative lung from contamination and do not protect the non-operative lobe(s) of the operative lung. Recent case reports have explored the protection of non-operative lobe(s) of the operative lung using solutions such as a DLT + BB or the double-bronchial blocker (DBB) technique^3–5^. These plans are feasible and can provide lung protection for nonsurgical lung lobes. However, given the small sample sizes of these previous reports, the feasibility and efficacy of using DLT + BB or DBB techniques in thoracoscopic lobectomy still need to be determined. Compared with using DLT, using BB showed more infiltrate especially at the surgery side based on the chest X-ray^6^. Therefore, we hypothesised that a DBB used with continuous positive airway pressure (CPAP) technology might protect the non-operative lobe(s) of the operative lung during thoracoscopic lobectomy.

This randomised controlled trial aimed to compare the effects of DBB with CPAP technology and those of routine BB on the non-operative lobe(s) of the operative lung and the incidence of postoperative pneumonia in both the operative and non-operative lung after thoracoscopic lobectomy.

## Methods

### Ethics

This study was approved by the Medical Ethics Committee of the Fourth Hospital of Hebei Medical University on 20 September 2021 (ID:2021116) and was registered before patient enrolment in the Chinese Clinical Trial Registry (http://www.chictr.org.cn; registration number: ChiCTR2100052086; Principal investigator: Jianfeng Fu; Date of registration: 16 October 2021). The trial was conducted from 1 November 2021 to 28 February 2022. Written informed consent was obtained from all patients before the trial. The first author of this article was responsible for recruiting participants among patients scheduled for video-assisted thoracoscopic lobectomy at our hospital. The manuscript was written in accordance with the CONSORT statement. The authors declare that all experiments on human subjects were conducted in accordance with the Declaration of Helsinki and that all procedures were carried out with the adequate understanding and written consent of the subjects.

### Participants

We recruited patients with lung cancer who were scheduled to undergo thoracoscopic lobectomy at the Fourth Hospital of Hebei Medical University. The inclusion criteria were as follows: (1) ASA grade I–II; (2) age 18–65 years, with a body mass index of 18.5–25 kg/m^2^; (3) no obvious abnormalities in pulmonary function, unlimited or obstructive ventilatory dysfunction, and FEV_1_/FVC > 70%; (4) no obvious abnormalities in cardiac function, no cardiovascular disease, and ejection fraction > 50%; (5) no preoperative anaemia or other haematological diseases and no history of radiotherapy or chemotherapy; and (6) voluntary study participation with provision of written informed consent. The exclusion criteria were as follows: (1) history of bronchial asthma and airway hyperreactivity; (2) pulmonary infection, bronchopleural fistula, emphysema, or pulmonary vesicles; and (3) presence of a tumour in the airway affecting the placement of bronchial blockers.

Using a computer-generated randomisation table, patients were randomly divided into two groups: the DBB with CPAP group (Group DBB; n = 40) and the routine BB group (Group BB; n = 40). The anaesthesiologists and surgeons, but not the postoperative follow-up team, were informed of the group allocation.

### Anaesthesia

After the patient entered the operative room, the nurse established upper limb venous access; conducted electrocardiography, pulse oximetry, non-invasive blood pressure measurement, and bispectral index monitoring; and performed radial artery puncture and catheterisation under local anaesthesia for invasive monitoring of arterial blood pressure. Anaesthesia was induced with sufentanil 0.2–0.4 μg/kg, cis-atracurium 0.2 mg/kg, and etomidate 0.2–0.3 mg/kg. After the eyelid reflex disappeared, mask-assisted ventilation was provided for 3 min.

**Figure 1:**
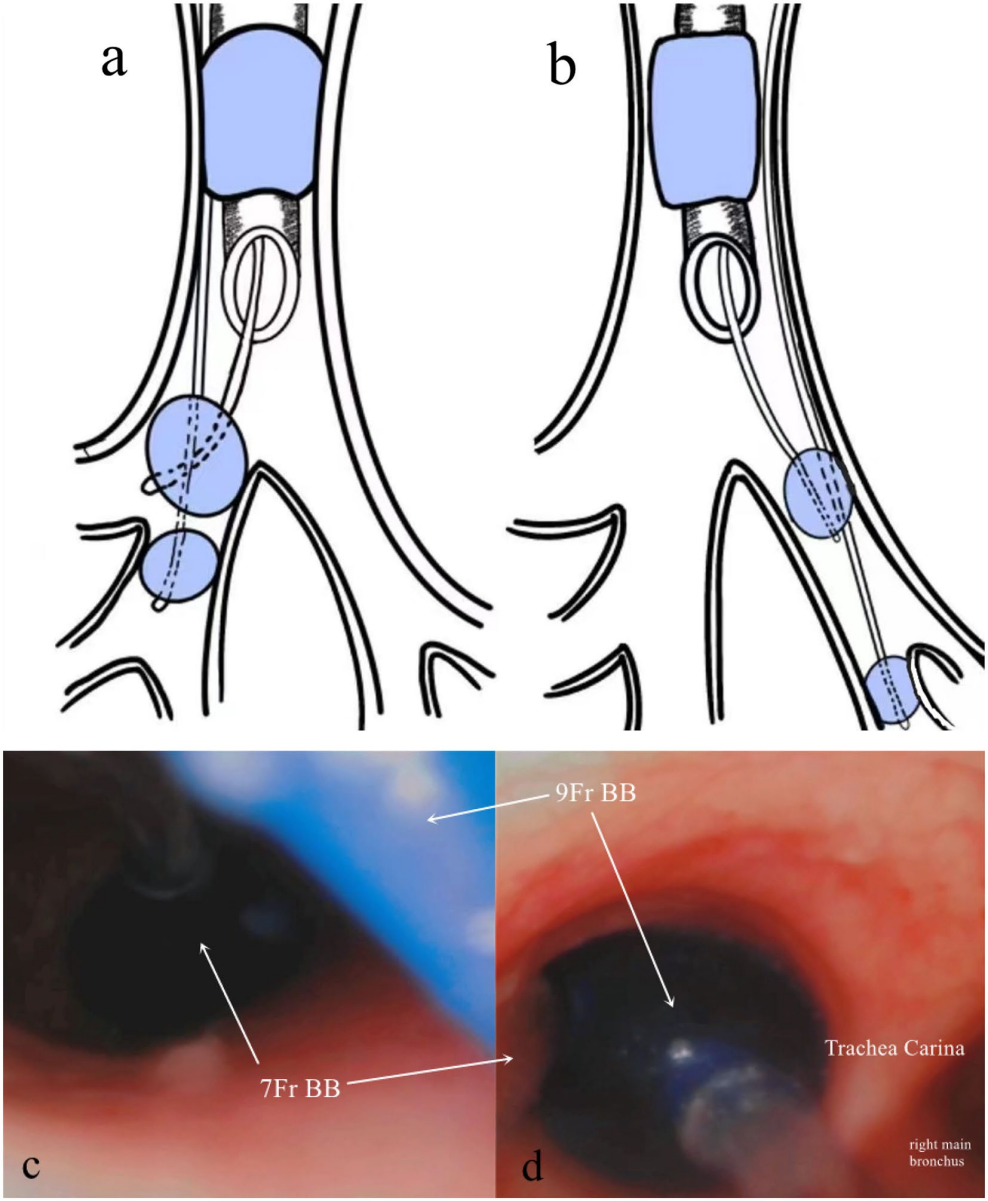
Position of the BB, (**a**): for right lung surgery, 7-Fr BB was placed in the middle bronchus of the right lung while the 9-Fr BB was placed through the tube in the right main bronchus; (**b**): for left lung surgery, 7-Fr BB was placed in the inferior lobar bronchus of the left lung while the 9-Fr BB was placed through the tube in the left main bronchus; (**c**, **d**): for left lung surgery, position of 7-Fr BB and 9-Fr BB in the patient's trachea.

The airway management protocol for patients in Group DBB was as follows. First, we inserted a 7-Fr BB through the mouth under direct vision. Next, we inserted a #8.0 tube 2–3 cm below the glottis. The position of the BB was adjusted using a fibre-optic bronchoscope (for right lung surgery, we placed the 7-Fr BB in the middle bronchus of the right lung; for left lung surgery, we put the 7-Fr BB in the inferior lobar bronchus of the left lung. We then fixed the tube and 7-Fr BB. Subsequently, a 9-Fr BB was put through the tube in the main bronchus of the operative lung) (Fig. 1). When starting single-lung ventilation, we provided 5 cm H_2_O CPAP to the nonsurgical lung lobe on the surgical side through the suction hole of the BB. This was maintained until the restoration of bilateral pulmonary ventilation.

The airway management protocol for the Group BB was as follows. First, an #8.0 tube was inserted through the mouth and placed 2–3 cm below the glottis. Next, a 9-Fr BB was put into the tube. A fibre-optic bronchoscope was used to confirm that the BB was in a good position and placed appropriately in the main bronchus of the operative lung. During one-lung ventilation, the BB suction port was connected to the atmosphere.

The anaesthesia maintenance and recovery methods were the same for all patients. We added 0.05 mg/kg cis-atracurium every half hour. The pressure-control ventilation mode was based on the ideal body weight, and dual-lateral ventilation was performed before the thoracic cavity was connected to the atmosphere. The respiratory parameters were as follows: tidal volume, 8–10 mL/kg; respiratory rate, 12–14 bpm; inspiration–expiration ratio, 1:2; inhaled oxygen concentration, 100%; and partial pressure of end-tidal carbon dioxide (PetCO_2_) maintained at 35–45 mm Hg. A fibre-optic bronchoscope was inserted with the patient lying on their side. When the pleura was opened, lung collapse was induced using a disconnection technique^7,8^. After manual recruitment of the non-operative lungs, one-lung ventilation was maintained. During one-lung ventilation, the respiratory parameters were as follows: tidal volume, 6–8 mL/kg; respiratory rate, 13–15 bpm; inspiration–expiration ratio, 1:2; inhaled oxygen concentration, 100%; PetCO_2_ maintained at 35–45 mm Hg. After surgery, the BB was removed, and the sputum was thoroughly suctioned. After spontaneous breathing was restored, the tracheal tube was removed, and the patient was returned to the ward after attaining a Steward score of 6.

### Outcomes

The primary outcome was the incidence of postoperative pneumonia in the operative and non-operative lungs on the first day after surgery. Three of the following five characteristics were pneumonia^9^: chest radiograph with infiltrate, fever > 38 °C, leucocytosis, antibiotic treatment, and positive sputum culture. Secondary outcomes included the duration of surgery, the incidence of hypoxaemia, BB displacement rate, surgeon satisfaction, white blood cell count (WBC), neutrophil count (N), neutrophil percentage (N%), C-reactive protein (CRP) level, length of hospital stay (LOS), and total hospital costs.

Based on the criteria above, the respiratory physician in the postoperative follow-up team determined whether the patient developed pneumonia. Hypoxaemia was considered to have occurred when oxygen saturation was < 90%. Intraoperatively, if a sudden increase in airway pressure occurred and ventilation could not be maintained, or if the operative lung suddenly inflated, we considered that BB displacement had occurred; accordingly, a bronchoscope was used to reposition the BB. Such occurrences were recorded.

Surgeon satisfaction with respect to the operating space was evaluated by the surgeon after the operation (Level I: large operating space, uneventful surgery; Level II: sufficient operating space to allow surgery completion; Level III: small operating space impeding surgery completion without adjustment).

### Statistical analysis

The incidence of pulmonary complications after thoracic surgery is high^10^, ranging from 10.7 to 50%^9,11–13^. Therefore, we assumed that the incidence of pneumonia in Group BB would be 45%. In a pilot study, the incidence of pneumonia in a DBB-treated group was approximately 15%. The formula for comparing the rates between two independent samples was as follows:

$$n=0.5\times {\left[\frac{({u}_{\alpha }+{u}_{\beta })}{{{\text{sin}}}^{-1}\sqrt{{p}_{1}}-{{\text{sin}}}^{-1}\sqrt{{p}_{2}}}\right]}^{2}$$

Accordingly, 35 participants were required in each group (power: 0.8; type I error: 0.05; ${u}_{\alpha }$ = 1.96; ${u}_{\beta }$ = 0.842). To address exclusion and loss, we increased the number of patients in each group and eventually targeted 40 patients per group.

Fisher's exact test was used for between-group comparisons of the incidence of postoperative pneumonia, expressed as a percentage. The *t*-test was used for between-group comparisons of the duration of surgery, WBC count, N count, N%, CRP, LOS, and total hospitalisation costs, with the results being expressed as mean ± standard deviation. Chi-square tests were used for between-group comparisons of the incidence of hypoxaemia, the BB displacement rate, and surgeon satisfaction. Statistical significance was set at *p* < 0.05. All analyses were performed using IBM SPSS Statistics 23.0 for Windows (IBM Corporation, Armonk, NY, USA).

## Results

**Figure 2:**
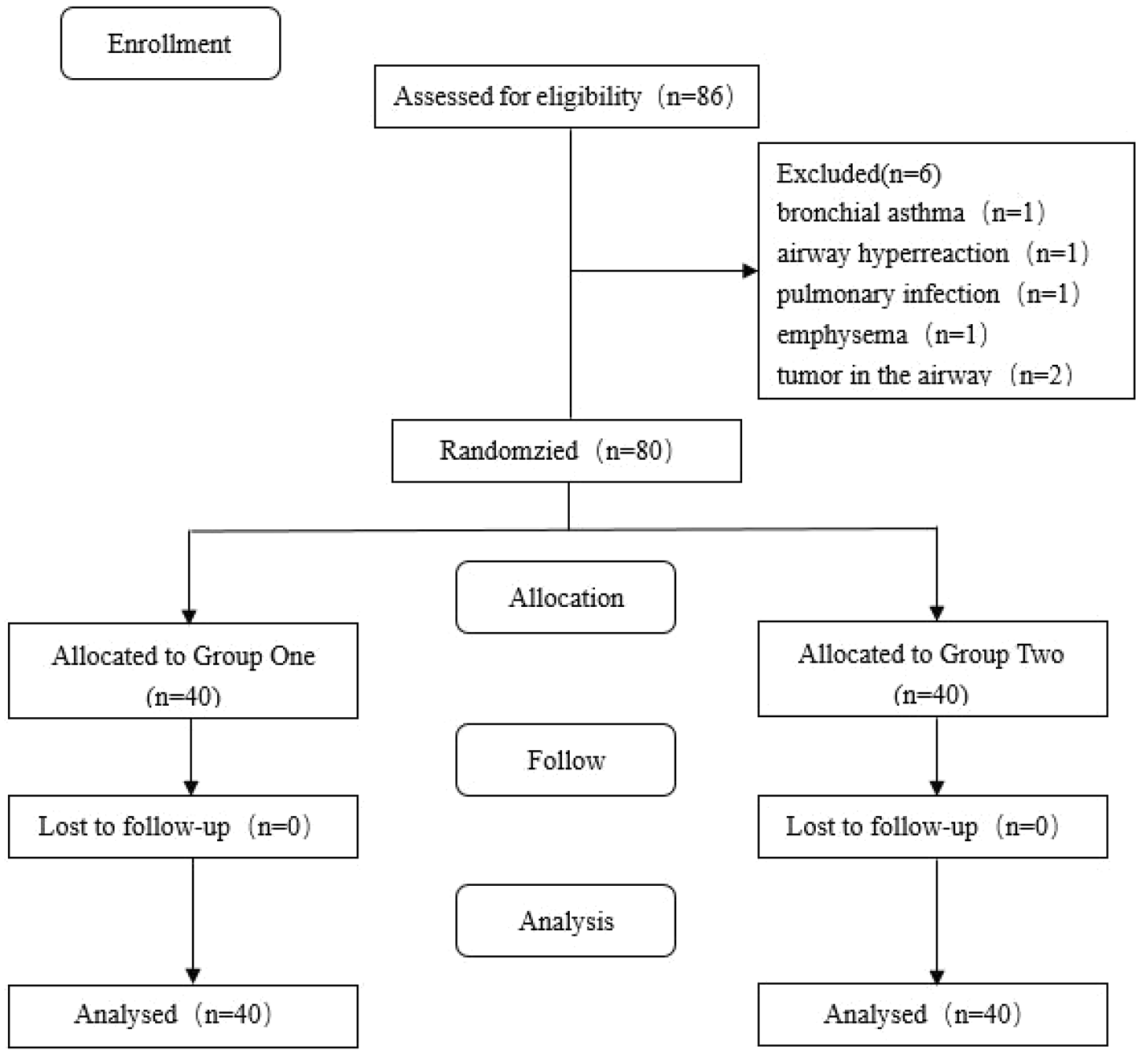
CONSORT diagram of the study participants.

Between November 2021 and February 2022, 80 patients were randomly and equally assigned to the two groups (Fig. 2). All the patients completed the trial. Table 1 presents the demographic characteristics of patients in each group.

**Table 1:** Characteristics of patients in the two groups.

	Group BB (n = 40)	Group DBB (n = 40)	*P* value
Age, years	62.33 ± 9.55	62.10 ± 6.42	0.902
Sex (male/female)	12/28	15/25	0.820
Height, cm	164.33 ± 7.6	164.58 ± 6.5	0.875
Weight, kg	59.98 ± 7.49	59.75 ± 7.28	0.886
ASA physical status			
I	11	10	1.000
II	29	30	
Operative side (left/right)	11/29	15/25	0.474

Compared with Group BB, Group DBB had a significantly lower incidence of postoperative pneumonia in the operative lung (*P* = 0.013; Table 2) and non-operative lung (*P* = 0.023; Table 2).

**Table 2:** The incidence of pneumonia on the first day after surgery in the two groups.

	Group BB (n = 40)	Group DBB (n = 40)	Difference (95% CI)	Risk ratio (95% CI)	P value
Operative lung	11 (27.5%)	2 (5%)	6.31%–38.26%	5.5 (1.30–23.25)	0.013
Non-operative lung	16 (40%)	6 (15%)	5.39%–42.33%	2.67 (1.16–6.11)	0.023

CI, confidence interval.

There were no significant between-group differences in the duration of surgery, the incidence of hypoxaemia, BB displacement rate, and surgeon satisfaction (*P* > 0.05; Table 3). Further, no significant between-group differences existed in the WBC count, N, N%, and CRP levels on the first day after surgery (*P* > 0.05, Table 3). Moreover, there were no significant between-group differences in hospitalisation time or total hospitalisation costs (*P* > 0.05, Table 3).

**Table 3:** Secondary outcomes in the two groups.

	Group BB (n = 40)	Group DBB (n = 40)	Difference (95% CI)	Risk ratio (95% CI)	P value
Duration of surgery (min)	166.55 ± 46.87	154.80 ± 62.55	11.75(− 12.85–36.35)	/	0.345
Hypoxemia	0	0	/	/	/
Displacement of BB	8 (20%)	9 (22.5%)	− 15.43%–20.26%	0.556–1.533	0.785
Surgeon’s satisfaction					
1	39	36	/	/	0.346
2	1	3			
3	0	1			
WBC (10^9^/L)	11.58 ± 3.23	10.90 ± 2.37	0.68(− 0.58–1.94)	/	0.288
N (10^9^/L)	9.71 ± 3.17	9.09 ± 2.42	0.62(− 0.64–1.87)	/	0.957
N% (%)	81.71 ± 8.68	81.61 ± 6.29	0.09(− 3.28–3.47)	/	0.333
CRP(mg/L)	39.19 ± 13.99	42.15 ± 12.92	− 2.96(− 8.96–3.03)	/	0.832
LOS (days)	6.80 ± 3.65	6.15 ± 2.36	0.65(− 0.72–2.02)	/	0.203
Hospitalisation cost (RMB)	76,733 ± 13,304	77,263 ± 14,972	− 530(− 6835–5774)	/	0.296

## Discussion

Postoperative pneumonia is a common complication of thoracoscopic lobectomy and can affect patient prognosis^9^. Our findings showed that the incidence of operative lung pneumonia in group BB was 5.5 times higher than in Group DBB. In contrast, the incidence of non-operative lung pneumonia in Group BB was 2.67 times higher than in Group DBB. These findings indicate that the use of a DBB significantly reduced the incidence of postoperative pneumonia, which could affect patient prognoses.

The two most commonly used airway tools for lung isolation are DLT and BB^14^, which are designed to prevent contamination of the non-operative lung by tumours or secretions from the operative lung^15^. However, these two airway tools cannot isolate the operative lobes from the non-operative lobes of the operative lung. In this study, we used the DBB technique to isolate not only the operative lung from the non-operative lung but also the operative lobe(s) from the non-operative lobe(s) of the operative lung, which prevented contamination of the non-operative lobe(s) by the operative lobe(s) of the operative lung. This may explain the low incidence of operative lung pneumonia in Group DBB. We provided the non-operative lobes of the operative lungs with 5 cm H_2_O CPAP through the BB suction hole. Studies have confirmed that 5 cm H_2_O CPAP does not affect the surgical field of view^16^ and that this can reduce the local immune response in the non-operative lobe(s) of the operative lungs^17^. This may be another reason for the relatively lower incidence of operative lung pneumonia in Group DBB.

During one-lung ventilation, hypoxic pulmonary vasoconstriction (HPV) causes blood flow to shift from the operative lung to the non-operative lung^16,18^, which increases blood flow in the non-operative lung by 49–56%^17^. High perfusion in the non-operative lung is directly related to lung injury^19^. A previous study confirmed that, during one-lung ventilation, there was a higher incidence of alveolar and interstitial oedema, haemorrhage, neutrophil infiltration, and microatelectasis in the non-operative lung than in the operative lung^19^. Similar conclusions have been drawn from different animal models. Broccard et al. suggested that the intensity of lung perfusion contributes to ventilator-induced lung injury^20^. A study on acute lung injury after lobectomy found that density increased more in the non-operated lung than in the operated lung, suggesting a truly asymmetric form of acute respiratory distress syndrome^21^. This explains the higher incidence of pneumonia in the non-operative lung than in the operative lung in both groups. In this study, we administered 5 cm H_2_O CPAP to the non-operative lobe(s) of the operative lung in Group DBB, which weakened HPV, attenuating the increase in operative lung vascular resistance and diminishing the transfer of blood flow from the operative lung to the non-operative lung^17,18^. This may be another reason for the lower incidence of non-operative lung pneumonia in Group DBB than in Group BB.

A high displacement rate and interruption of surgery have been reported as disadvantages of BB^22^. We observed no significant between-group difference in the BB displacement rate, consistent with previous reports^22–24^. The BB displacement rate is related to the placement position; because the right main bronchus is shorter, the BB displacement rate in right lung surgery is higher than that in left lung surgery^25^. In Group DBB, one BB was placed deep in the inferior lobar bronchus of the left lung or middle lobar bronchus of the right lung, which made it less likely to shift. There were no cases of hypoxaemia; further, there were no significant between-group differences in surgeon satisfaction, indicating that the minor expansion of the non-operative lung lobe did not affect the execution of the surgery, and it did not cause hypoxaemia because of BB displacement.

Numerous factors affect patient prognosis and LOS, such as patient age and the extent of surgical resection^10^. In this study, we used randomisation to allocate patients to groups, with no significant between-group differences in age or surgical lung tissue. We reduced the incidence of postoperative pneumonia in patients who underwent DBB through the indicated intervention. Although the LOS in this group was 0.5 d shorter than that in Group BB, this difference did not reach statistical significance. The sample size of this trial was estimated based on the incidence of postoperative pneumonia; therefore, the sample size may have been too small to identify significant differences in LOS. Future tests with larger sample sizes are required.

To ensure the stability of CPAP, we used a device that could continuously adjust the pressure and monitor CPAP in real-time using a pressure sensor to ensure that the pressure applied was the pressure that was set for the experiment. In Group DBB, one patient experienced inflation of the non-operative lobe(s) of the operative lung because of CPAP pressure of 5 cm H_2_O, which affected the surgeon's ability to operate. The surgery could be performed smoothly after adjusting the CPAP pressure to 4 cm H_2_O. Currently, most research on CPAP is conducted using a DLT; moreover, it has been concluded that a CPAP of 5 cm H_2_O can improve oxygenation^26,27^. Few studies have investigated the optimal CPAP administered by BB, with only a few case reports using a pressure of 5 cm H_2_O^28,29^; therefore, we used a CPAP of 5 cm H_2_O in this study.

## Limitations

This study had some limitations. First, it was a single-centre study. To ensure consistency among the study participants, the participants selected for this study underwent lobectomy and did not undergo procedures such as pneumonectomy. Therefore, it remains unclear whether DBB reduces the incidence of pneumonia in patients undergoing other surgical procedures. Second, the sample size was estimated based on the incidence of pneumonia in patients, which was relatively small. Finally, the anaesthesiologists and surgeons could not be blinded. Because the surgeon can see the location of the blue cuff of the BB under the thoracoscope, it was easy for the surgeon to know the patient's group allocation. Additionally, two BBs were used in our study, increasing the patient burden. Using an improved BB to achieve the effects of the DBB technique in subsequent studies may be more beneficial to patients.

## Conclusions

In conclusion, the current study showed that, for thoracoscopic lobectomy, using the DBB technique to isolate the operative from the non-operative lobe(s) of the operative lung and providing CPAP to the non-operative lobe(s) through the BB can reduce the incidence of postoperative pneumonia in both lungs. This does not increase the rate of BB displacement or complicate the execution of the surgery and holds benefits for patient prognosis. Further larger-scale studies on this topic should investigate the extent of the benefits this approach has for patients.

### Supplementary Information

Supplementary Information.

## Abbreviations

DBB: Double-bronchial blocker
CPAP: Continuous positive airway pressure
BB: Bronchial blocker
DLT: Double-lumen tubes
WBC: White blood cell count
N: Neutrophil count
N%: Neutrophil percentage
CRP: C-reactive protein
LOS: Length of hospital stay
PetCO_2_: Pressure of end-tidal carbon dioxide
HPV: Hypoxic pulmonary vasoconstriction
